# Quality of life in the general population of Mongolia: Normative data on WHOQOL-BREF

**DOI:** 10.1371/journal.pone.0291427

**Published:** 2023-09-29

**Authors:** Enkhjin Bat-Erdene, Tetsuya Hiramoto, Enkhnaran Tumurbaatar, Gantsetseg Tumur-Ochir, Otgonbold Jamiyandorj, Eiko Yamamoto, Nobuyuki Hamajima, Takakazu Oka, Tsolmon Jadamba, Battuvshin Lkhagvasuren

**Affiliations:** 1 Department of Neuroscience and Cell Biology, Child Health Institute of New Jersey, Robert Wood Johnson Medical School, Rutgers University, New Brunswick, New Jersey, United States of America; 2 Brain Science Institute, Graduate School, Mongolian National University of Medical Sciences, Ulaanbaatar, Mongolia; 3 Brain and Mind Research Institute, Mongolian Academy of Sciences, Ulaanbaatar, Mongolia; 4 Department of Psychosomatic Medicine, Fukuoka National Hospital, National Hospital Organization, Fukuoka, Japan; 5 Department of Mental Health Surveillance, National Center for Mental Health, Ulaanbaatar, Mongolia; 6 Department of Healthcare, Ulaanbaatar City Government, Ulaanbaatar, Mongolia; 7 Department of Healthcare Administration, Nagoya University Graduate School of Medicine, Nagoya, Japan; 8 Kishokai Medical Corporation, Nagoya, Japan; 9 Department of Psychosomatic Medicine, International University of Health and Welfare Narita Hospital, Narita, Japan; Tehran University of Medical Sciences, ISLAMIC REPUBLIC OF IRAN

## Abstract

No data on the quality of life (QOL) of the general population are available for Mongolia. This study aimed to determine normative data on the World Health Organization Quality of Life-Brief Version (WHOQOL-BREF) in the general population of Mongolia. This nationwide, population-based, cross-sectional study was conducted in 48 sampling centers across Mongolia in 2020. We used the WHOQOL-BREF and the Hospital Anxiety and Depression Scale (HADS) in our study and evaluated their associations with vital signs, body measurements, and lifestyle determinants. A total of 714 participants (261 men and 453 women) with a mean (standard deviation) age of 40.7 (13.2) years were recruited. The mean scores of WHOQOL-BREF subscales were 61.5 for physical health, 73.5 for psychological health, 70.1 for social relationship, and 67.2 for environmental health domains. The prevalence of poor QOL was 16.9% among the participants. Participants living in an apartment in urban areas with high HADS scores had a low QOL. All domains of WHOQOL-BREF were inversely correlated with anxiety score (r = -0.353 ‐ -0.206, p < 0.001) and depression scores (r = -0.335 ‐ -0.156, p < 0.001). Physical health was predicted by residency location, anxiety, and depression (R^2^ = 0.200, p < 0.001); psychological health by anxiety and depression (R^2^ = 0.203, p < 0.001); social relationship by residency location, age group, anxiety and depression (R^2^ = 0.116, p < 0.001); and environmental health by employment, anxiety, and depression (R^2^ = 0.117, p < 0.001). This is the first report on normative data on the QOL in the general population of Mongolia. Physical health was low compared with that determined using international data. Poor QOL was observed among those with mental health issues living in the urban areas.

## Introduction

Mongolia has a small population but a large geographical area. More than half of the population lives in rural areas as nomadic herders in a traditional pelt tent called a ger [1]. Gers are not provided with central electricity, water, sanitation, or heating systems. Conversely, urban residents who predominantly reside in apartments benefit from a connection to centralized utilities, including winter heating systems. Notably, a significant proportion of these structures were constructed several decades ago, adhering to Soviet-era standard panel building designs [2]. A large part of the country is arid and extremely cold, with January averages dropping as low as -30°C. The capital city, Ulaanbaatar, makes up most of the urban areas, whereas prefecture centers play marginal roles because of the lack of infrastructure required for modern cities. Ulaanbaatar is the coldest capital city in the world, with an annual average temperature of -1°C [3]. The rural areas are divided into four regions: eastern, western, mountain (Khangai), and central. Each region has three to seven geopolitical prefectures. Since 1990, after the democratic revolution, the rural population has migrated to Ulaanbaatar, surpassing a million residents. This migration has expanded ger areas in Ulaanbaatar making it one of the most air-polluted cities in the world [4]. While some family households within Ulaanbaatar’s ger areas possess conventional houses, comprehensive central utility systems remain absent, with the exception of electricity. Consequently, living conditions in Mongolia diverge significantly from those commonly perceived in Western countries.

Because of the politico-economical changes, Mongolia’s disease burden has shifted from communicable to non-communicable diseases [5]. This rapid alteration in disease burden might have affected younger people more than older residents, similar to other developing countries [6]. To meet the shift in disease burden, Mongolia has to establish appropriate policies to improve the efficacy of healthcare services by monitoring the quality of life nationwide (QOL). The use of a QOL assessment instrument is generally recommended for evaluating the effectiveness of preventive, diagnostic, and treatment measures.

Notably, no studies have assessed the QOL of the general population in Mongolia. In particular, the political transition from communism to democracy, rapid urbanization, air pollution, lifestyle changes, the shift in disease burden, and economic turbulence over the past 3 decades should have largely impacted the QOL of the Mongolian people. Including these factors, the subjective perception of QOL among Mongolian people may deviate from international standards.

QOL assessment instruments, such as the Short-Form Health Survey (SF-36), EuroQOL (EQ-5D), and World Health Organization Quality of Life (WHOQOL-100), have been examined in terms of validity and reliability across different cultures. Among them, the WHOQOL-100 was developed by WHO experts with simultaneous consideration of the context of the cultural aspects in 15 international field centers. The abbreviated version, the brief version of the World Health Organization Quality of Life (WHOQOL-BREF), is a concise self-report questionnaire that assesses health-related QOL in both general and clinical populations. It consists of 24 Likert-scale items representing four latent domains: physical health, psychological health, social relationships, and environmental health. Two additional exclusive questions from the measurement model estimate the participant’s satisfaction with their life and general health [7]. We translated and determined the psychometric properties of WHOQOL-BREF in the general population of Ulaanbaatar in our previous study [8]. Our results demonstrate that the Mongolian version of WHOQOL-BREF has good validity and reliability for assessing QOL in the general population. Using the same tool, this study aimed to establish normative data on QOL in the Mongolian population. Furthermore, sociodemographic, physical, and psychological characteristics of the Mongolian population have been extensively investigated to determine factors associated with the QOL.

## Materials and methods

### Study participants

This study was part of a nationwide, multicenter, interdisciplinary, prospective, population-based cohort study that investigated brain-related disorders in the general population of Mongolia by the Brain Science Institute at the Mongolian National University of Medical Sciences.

**Fig 1:**
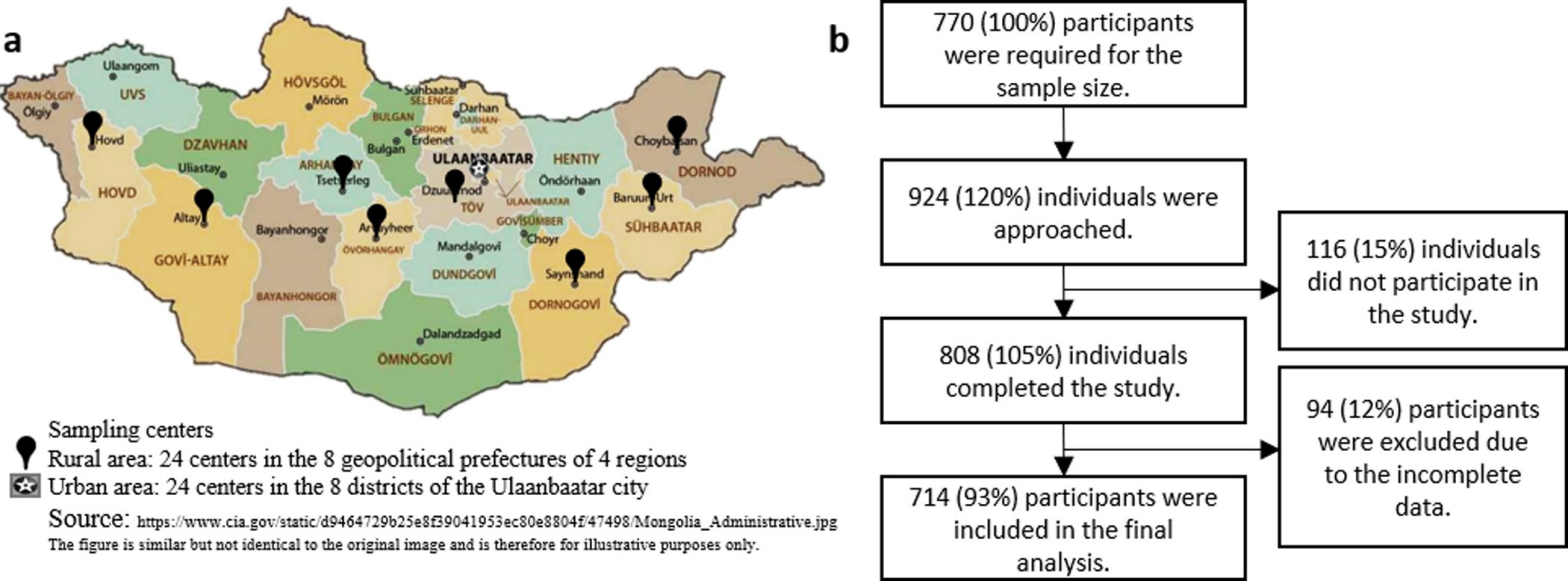
Study flowchart and sampling sites. a) Sampling sites across the country. The study consisted of 48 sampling centers, including 24 in 8 districts of Ulaanbaatar and 24 in 8 prefectures of 4 rural regions. b) Study flowchart: The required sample size was 770. We invited 924 individuals to participate in this study. Among them, 116 refused, and 94 participants were excluded due to the missing data. A total of 714 participants were included in the final analysis.

In this cross-sectional study, the sample size was calculated to be 385 for a population size of 1,910,630 individuals aged 18–65 years in Mongolia [1]. According to the 2020 census, 47% of the population lived in the capital city of Ulaanbaatar, and the remaining lived in four rural regions. To cover a full representative population of the rural regions, we included two residency locations (urban and rural areas), resulting in the desired sample size of 770. Considering a response rate of 80%, 924 individuals who fulfilled the inclusion criteria were invited to participate in the study. Mongolian citizens who lived in geopolitical units for at least 6 months were considered to meet inclusion criteria. The participants were recruited from 48 sampling centers, including 24 primary health centers in 8 districts in Ulaanbaatar and 24 primary health centers in 8 prefectures of 4 rural regions across the country (Fig 1A).

The sampling centers were located at primary health centers where the entire population was registered six age-sex groups (18–29, 30–44, 45–65 years; men and women) to create a representative sample that matches the age and sex distribution of the population. Depending on the population density, two or three individuals for each age-sex group from a sampling center were randomly selected using a computer program. Among the invited individuals, 116 did not reach the sampling center on a given date and time. A total of 808 participants completed the survey, and 94 of whom had missing data. The remaining 714 participants were included in the final analysis (Fig 1B).

### Data collection

The data collection started on September 7, 2020, and was completed on November 29, 2020. The study was conducted in the official language (Mongolian). Trained research personnel or medical doctors explained the WHOQOL-BREF questionnaire to the participants face-to-face and helped them to respond using a tablet. In addition, demographic characteristics and lifestyle data were collected. Height, weight, waist circumference, and neck circumference were also measured. To determine the current physical health status, four primary vital signs were examined noninvasively by trained research personnel or medical doctors: body temperature at the forehead or wrest with an electronic infrared thermometer (Tida, TD-133, China), blood pressure and heart rate using an advanced blood pressure monitor (BP A6 PC, Microlife, Switzerland), and arterial oxygen saturation (SpO2) using a pulse oximetry (PO40, Beurer, Germany). Blood pressure, heart rate, and arterial oxygen saturation were assessed in accordance with the WHO’s guidelines on the measurement of these vital signs [9,10].

### Instruments

The WHOQOL-BREF), one of the most commonly used generic QOL questionnaires, was developed by the WHOQOL group in 1996 [11]. The WHOQOL-BREF is open-source, and accessible for non-commercial use, and translated into more than 40 languages. This method is suitable for large-sample surveys and clinical trials. This 24-item questionnaire determines QOL using four domain scores: physical health (questions 3,4,10,15–18), psychological health (questions 5–7,11,19,26), social relationships (questions 20–22), and environmental health (questions 8–9, 12–14, 23–25). Each item is measured on a 5-point Likert-scale. The score of each domain consists of the mean score of items multiplied by four, in which a higher score indicates a better QOL. Each domain score is converted to a scale of 0–100. Two more items on the perception of overall QOL and general health were aggregated to the general facet, which was also converted to a scale of 0–100 [12]. We translated the WHOQOL-BREF into Mongolian based on the cross-cultural adaptation guideline and described its psychometric properties in our previous study [8]. The translated version showed a good fit for validity and reliability in the general population. To confirm the previous results, we calculated the Cronbach’s alpha coefficients for all domains of the WHOQOL-BREF. Cronbach`s alpha was 0.84 for the total scale, and 0.84, 0.79, 0.81, and 0.76 for the physical health, psychological health, social relationship, and environmental health domains, respectively.

The Hospital Anxiety and Depression Scale (HADS) is a 14-item self-report questionnaire widely used to evaluate the severity of anxiety and depression symptoms in the past week. It was developed to identify mental symptoms, including anxiety and depression, in the general population and patients in clinical settings [13]. Among the 14 items, 7 were for anxiety (HADS-A subscale), and the remaining 7 were for depression (HADS-D subscale) formulated in a readily understandable language. Each item was rated on a 4-point scale from 0 to 3 for a total score between 0 and 21 for each subscale. The ranges of scores for cases on each subscale were 0–7, normal; 8–10, mild abnormality; 11–14, moderate abnormality; and 15–21, severe abnormality. The HADS has been translated into Mongolian and has good psychometric properties [14].

### Statistical analysis

Data were presented as mean ± standard deviation (SD). The distributions of continuous variables were evaluated by Shapiro-Wilk test. Differences between the two groups were examined using the χ^2^ test for categorical data and Mann-Whitney U-test for continuous data. To determine differences in the domain scores between sociodemographic characteristics for each group (rural vs. urban areas; gers vs. apartments), a one-way ANOVA or t-test was performed, as appropriate. To estimate the prevalence of poor QOL, cut-off points for each subscale domain of the WHOQOL-BREF were established by dichotomizing the domain score at 1 SD below the mean of domain score in the participants of this study [15]. Correlation analyses between continuous variables were performed using Spearman’s bivariate test. Multiple linear regression analyses with the backward stepwise method were used to determine if factors (independent variables: all variables, including sociodemographic characteristics, body measurements, vital signs, and HADS scores) were associated with the mean scores of each domain of the WHOQOL-BREF (dependent variables: physical health, psychological health, social relationship, and environmental health domains). In the residency location variable, we used capital city Ulaanbaatar as the reference group. The remaining four rural regions were combined into one category. Multicollinearity was examined using variance inflation factor (VIF) and tolerance (1 < VIF < 2.5; tolerance < 10). Homoscedasticity was assessed using scatter plots of residuals by predicted values. No outliers were detected (Cook’s distance, < 1; standard residuals < ±3.3). The independence assumption was tested using the Durbin–Watson coefficient (satisfied if 1.5 < Durbin–Watson < 2.5). To construct a receiver operating characteristic (ROC) curve, the general facet variable was dichotomized at 1 SD below the mean score. Using the new nominal variable, we evaluated the screening ability of the WHOQOL-BREF domains at a range of cut-off points.

All statistical tests were two-tailed with a statistical significance set at p < 0.05. Data were analyzed using SPSS v26.0 and JAMOVI v2.2.5.

### Ethical considerations

Written informed consent was obtained from all participants. The Institutional Review Board and Ethics Committee of the Mongolian National University of Medical Sciences (MNUMS) approved the study protocol and procedures for obtaining informed consent (number: 2020/03-05).

## Results

There were 714 participants (261 men and 453 women) aged 18–65 years, with a mean ± SD of 40.7 ± 13.2 years. The details of the sociodemographic characteristics are described in Table 1. Because the participants had more females and were older, we adjusted for age and sex for further analyses by weighting them with the population report from the 2020 Population and Housing By-Census of Mongolia. The descriptive results suggested that the sample population represented the general population of the country (S1 Table). There were no missing data, except for 91 and 108 participants who did not report alcohol and tobacco use, respectively.

**Table 1:** Sociodemographic characteristics of the participants by sex.

Characteristics, n (%)		Total	Male	Female	p value^*^
Total		714 (100)	261 (100)	453 (100)	
Age group	18–29	181 (25.4)	52 (19.9)	129 (28.5)	**< 0.001**
	30–44	248 (34.7)	83 (31.8)	165 (36.4)	
	45–65	285 (39.9)	126 (48.3)	159 (35.1)	
Marital status	Never-married	119 (16.7)	50 (19.2)	69 (15.2)	0.526
	Others^#^	76 (10.6)	21 (8.0)	55 (12.1)	
	Married	519 (72.7)	190 (72.8)	329 (72.6)	
Education	Middle school and below	302 (42.3)	134 (51.3)	168 (37.1)	**< 0.001**
	Associate’s degree	186 (26.1)	61 (23.4)	125 (27.6)	
	Bachelor’s degree	193 (27.0)	56 (21.5)	137 (30.2)	
	Master’s degree and above	33 (4.6)	10 (3.8)	23 (5.1)	
Employment	Unemployed	88 (12.3)	24 (9.2)	64 (14.1)	0.545
	Student	84 (11.8)	35 (13.4)	49 (10.8)	
	Pensioner	140 (19.6)	59 (22.6)	81 (17.9)	
	Employed	402 (56.3)	143 (54.8)	259 (57.2)	
Income	< ₮500,000	434 (60.8)	154 (59.0)	280 (61.8)	0.295
	₮500,001 - ₮1,000,000	258 (36.1)	96 (36.8)	162 (35.8)	
	> ₮1,000,000	22 (3.1)	11 (4.0)	11 (2.4)	
Living condition	Ger (traditional pelt tent)	211 (29.6)	73.0 (28.0)	138 (30.5)	0.989
	House	223 (31.2)	88 (33.7)	135 (29.8)	
	Dormitory	42 (5.9)	15.0 (5.7)	27 (6.0)	
	Apartment	238 (33.3)	85 (32.6)	153 (33.8)	
Residency location	Eastern region	84 (11.8)	42 (16.1)	42 (9.3)	**0.001**
	Western region	107 (15.0)	52 (19.9)	55 (12.1)	
	Mountain region	110 (15.4)	36 (13.8)	74 (16.3)	
	Central region	101 (14.1)	24 (9.2)	77 (17.0)	
	Ulaanbaatar city	312 (43.7)	107 (41.0)	205 (45.3)	
Alcohol use	Yes	183 (29.6)	55 (28.1)	128 (30.3)	0.578
	No	436 (70.4)	141 (71.9)	295 (69.7)	
Tobacco use	Yes	127 (20.3)	55 (27.2)	72 (17.0)	**0.011**
	No	479 (76.6)	141 (69.8)	338 (79.9)	
	Had smoked before	19 (3.0)	6 (3.0)	13 (3.1)	
**Continuous variables, mean ± SD**					
Age		40.7±13.2	42.7±13.2	39.5±13.1	**0.002**
Vital signs	Body temperature	36.4±0.3	36.4±0.3	36.4±0.3	0.326
	Heart rate (per minute)	78.2±11.4	77.8±12.5	78.4±10.8	0.288
	Arterial systolic pressure	126.1±19.2	130.9±19.6	123.4±18.4	**< 0.001**
	Arterial diastolic pressure	80.4±12.9	83.6±13.0	78.5±12.5	**< 0.001**
	Arterial oxygen saturation	95.0±2.2	95.2±2.2	94.8±2.2	**0.030**
Body mass index (BMI)		26.9±5.5	27.3±5.4	26.7±5.5	0.161
Psychological symptoms (HADS score)	Anxiety	6.2±3.2	6.1±3.1	6.3±2.3	0.706
	Depression	5.8±2.1	5.8±2.7	5.8±2.9	0.633

^*^ p values were analyzed with the Chi-square test and Mann-Whitney *U*-test.

^#^ Others included remarried, co-habiting, separated, divorced, and widowed. ₮: Mongolian tugrik (MNT₮), US $1 = MNT ₮2850. HADS: Hospital Anxiety and Depression Scale. n: Number. SD: Standard deviation. There was no missing data, except for 91 and 108 participants who did not report alcohol and tobacco use, respectively.

There were no differences in marital status, employment, income, living condition, alcohol use, body temperature, heart rate, body mass index (BMI), and psychological symptoms between men and women. Women were more educated, lived more in urban areas, smoked less, and had less blood pressure. The average monthly expenditure was 215,000 Mongolian tugrik (₮) ($1 = ₮2850), and the minimum monthly income was ₮320,000. Of the participants, 60.8% received less than ₮500,000 (low income), 36.1% received ₮500,000–1,000,000 (middle income), and 3.1% received more than ₮1,000,000 (high income). Approximately 30% of the sample population lived in traditional gers. However, another 30% lived in houses that were usually not connected with water, sanitation, and heating system, except electricity.

Table 2 shows the mean and SD of each domain of the WHOQOL-BREF and general facet by sociodemographic characteristics among the 714 participants.

**Table 2:** Normative values of the WHOQOL-BREF scores by sociodemographic characteristics.

Characteristics (n)		WHOQOL domains (mean ± SD)				Perception
		PHY	PSY	SOC	ENV	GEN
Total, unadjusted (714)		61.5±12.6	73.6±12.1	70.4±15.9	67.6±13.4	70.5±15.1
Total, age and sex adjusted (714)		61.5±1.7	73.5±2.2	70.1±2.2	67.2±1.9	70.6±1.8
Male (261)	18–29 (52)	59.7±14.5	69.2±17.4	63.1±21.7	63.2±17.6	70.4±19.5
	30–44 (83)	63.5±13.3	76.4±11.7	73.7±15.6	67.8±13.6	72.4±13.5
	45–65 (126)	60.9±13.1	73.7±10.9	69.8±13.7	66.8±12.5	70.7±14.0
All male, age-adjusted (261)		61.6±4.0	73.4±5.1	69.4±5.4	66.1±4.3	71.3±4.3
Female (453)	18–29 (129)	61.8±13.2	73.0±13.0	73.3±17.6	69.4±14.3	72.5±15.7
	30–44 (165)	61.3±12.1	73.6±12.1	70.8±15.9	67.4±13.5	68.6±16.5
	45–65 (159)	61.3±11.2	73.7±9.9	68.7±12.8	68.3±11.2	69.5±12.9
All female, age-adjusted (453)		61.4±3.5	73.5±4.4	70.8±3.8	68.3±3.7	70.0±3.5
Marital status	Never-married (119)	60.4±13.1	71.1±13.4	68.3±17.3	66.2±14.8	72.1±15.4
	Others* (76)	62.5±10.8	75.7±2.3	72.7±13.3	69.7±9.8	70.1±13.1
	Married (519)	61.6±12.8	73.8±12.2	70.5±15.9	67.6±13.5	70.2±15.3
Education	Middle school and below (302)	62.5±12.3	74.1±12.5	70.7±15.2	68.1±13.2	71.4±15.6
	Associate’s degree (186)	61.7±12.2	73.8±11.1	71.5±14.3	67.9±12.8	70.2±14.2
	Bachelor’s degree (193)	59.8±13.6	72.2±12.6	68.2±18.3	66.6±14.4	69.4±15.6
	Master’s degree and above (33)	60.3±11.5	75.4±10.3	74.5±14.0	67.8±13.2	70.1±12.5
Employment	Unemployed (88)	60.4±12.4	72.9±11.3	70.6±15.7	66.4±14.3	70.2±15.2
	Student (84)	62.3±12.9	73.4±14.3	71.0±19.5	67.0±15.5	75.3±17.0
	Pensioner (140)	61.2±12.5	72.6±10.8	68.2±13.6	71.0±15.8	69.9±14.5
	Employed (402)	61.6±12.7	74.0±12.2	71.0±15.8	68.2±13.1	69.7±14.7
Income	< ₮500,000 (434)	61.4±12.9	73.4±12.0	70.0±16.0	67.5±13.6	71.1±15.2
	₮500,001 - ₮1,000,000 (258)	61.5±12.5	73.6±12.0	71.0±16.0	67.8±13.2	69.7±15.1
	> ₮1,000,001 (22)	61.4±10.4	75.2±14.2	70.8±13.5	67.0±11.7	67.0±13.1
Living condition	Ger (traditional pelt tent) (211)	63.4±12.9	75.0±10.7	71.3±14.0	69.3±12.5	71.1±13.6
	House (223)	61.1±12.4	73.4±11.9	71.0±15.6	68.0±13.4	69.7±15.0
	Dormitory (42)	62.5±13.2	74.5±14.6	71.6±19.9	66.8±13.5	78.3±18.1
	Apartment (238)	60.0±12.4	72.3±12.7	68.7±16.8	65.8±14.1	69.2±15.5
Residency location	Eastern region (84)	64.0±12.9	75.0±10.8	73.8±13.7	67.9±12.3	70.7±14.6
	Western region (107)	65.7±10.0	72.2±11.3	72.3±13.8	69.0±12.2	73.5±13.0
	Mountain region (110)	64.5±12.4	75.2±9.2	70.5±12.8	68.9±11.4	71.3±12.6
	Central region (101)	61.2±12.0	72.7±14.7	69.7±19.3	67.4±15.1	70.0±18.2
	Ulaanbaatar city (312)	58.4±12.9	72.4±12.5	70.4±15.9	66.6±14.1	69.2±15.5
Alcohol use	Yes (183)	60.7±13.8	73.4±12.3	69.3±18.4	67.0±13.9	68.8±14.8
	No (436)	61.6±12.4	73.5±12.1	70.8±14.7	68.0±13.2	70.9±15.2
Tobacco use	Yes (127)	62.3±12.7	73.4±13.4	71.1±16.2	68.8±13.8	70.9±16.4
	No (479)	61.3±12.5	73.6±11.7	70.4±15.5	67.6±12.9	70.4±14.5
	Had smoked before (19)	58.3±13.1	71.1±14.4	67.5±20.0	65.1±15.8	67.1±19.2
BMI ranges	Underweight (10)	64.3±10.9	78.3±6.1	75.8±12.7	71.3±12.0	71.3±20.5
	Normal weight (248)	61.8±13.1	73.0±13.4	70.5±17.0	67.7±13.7	70.8±16.2
	Pre-obesity (202)	60.7±12.2	74.1±11.3	70.8±15.1	67.8±12.8	70.4±13.5
	Obesity class I (114)	62.4±12.1	74.1±10.5	70.2±14.2	68.0±12.9	70.5±14.1
	Obesity class II (40)	60.6±12.5	70.7±12.9	69.2±17.2	67.3±15.0	68.4±15.0
	Obesity class III (13)	54.4±12.8	70.2±11.9	60.9±14.2	62.0±15.3	66.3±14.8
Anxiety level	Normal (482)	64.1±11.7	76.0±11.0	72.5±15.1	69.6±12.7	72.6±14.4
	Borderline normal (157)	56.7±13.0	70.3±12.0	66.6±16.1	63.4±13.0	67.0±15.3
	Abnormal (75)	54.5±12.1	64.8±13.2	64.8±17.6	63.3±15.8	64.2±16.4
Depression level	Normal (535)	63.6±11.5	75.7±10.4	72.4±14.5	69.2±12.2	71.7±14.3
	Borderline normal (137)	54.9±13.6	67.4±13.4	65.6±18.4	62.5±15.4	66.6±17.1
	Abnormal (42)	55.9±14.0	66.6±13.1	60.7±17.4	63.6±16.3	67.0±15.1

* Others included remarried, co-habiting, separated, divorced, and widowed. ENV: Environmental health domain. GEN: General facet. PHY: Physical health domain. PSY: Psychological health domain. SOC: Social relationship domain. There were no missing data, except for 91 and 108 participants who did not report alcohol and tobacco use, respectively.

The age- and sex-adjusted mean scores of the WHOQOL-BREF subscale domains were 61.5 for physical health, 73.5 for psychological health, 70.1 for social relationship, 67.2 for environmental health, and 70.6 for the general facet. There were no differences in the WHOQOL-BREF scores between sex, age group, education, income, alcohol use, tobacco use, and BMI ranges among the participants. Never-married participants had lower scores than those with different marital status in the psychological health domain (p = 0.022). Unemployed and pensioned participants had lower scores than the other participants in the general facet (p = 0.020). Participants living in apartments had lower scores than the other participants in the physical health domain and general facet (p = 0.039 and p = 0.003, respectively). Interestingly, subgroup analyses suggested that participants living in gers or houses without central utilities differ in the quality of life between residency locations only (rural vs. urban areas), whereas participants living in apartments with central utilities differed between age groups, sex, education, and employment (S2 Table). Participants living in Ulaanbaatar had lower scores in the physical health domain than those living in rural areas (p < 0.001). Furthermore, subgroup analyses suggested that participants living in Ulaanbaatar did not differ in the quality of life between sociodemographic characteristics (ger vs. apartment), whereas participants living in rural areas differed between age groups, marital status, and income (S3 Table). Participants with abnormal or borderline anxiety or depression had lower scores than those without anxiety or depression in all domains and general facet (all p < 0.001). Among the male participants, the scores of psychological health and social relationship domains peaked in the middle age (30–44 years) and then dropped (p = 0.007 and p = 0.001, respectively). Among female participants, the social relationship decreased as age increased (p = 0.040).

The prevalence of poor QOL for each domain had the following cut-off points using 1 SD below the mean criterion: 49 for physical health, 61 for psychological health, 54 for social relationship, and 54 for environmental health (Table 3). We found that 13.9–20.0% of the participants had a poor QOL (15.0–22.9% for men and 12.8–17.3% for women). There was no difference in the prevalence of poor QOL between the sexes. In contrast, the prevalence of poor QOL in psychological health differed between age groups. Younger participants had poorer psychological health than older participants.

**Table 3:** Prevalence of poor QOL by sex and age group.

Characteristics		WHOQOL-BREF domains							
		PHY		PSY		SOC		ENV	
		Count	%	Count	%	Count	%	Count	%
Cut-off scores using the 1 SD below the mean criterion (PHY 49; PSY 61; SOC 54; ENV 54)									
Male	18–29	11	26.8	14	38.9	35	16.6	17	34.5
	30–44	9	22.0	7	19.4	72	34.1	11	25.5
	45–65	21	51.2	15	41.7	104	49.3	22	40.0
	Total	41	15.7	36	13.8	50	19.2	55	21.1
Age-adjusted %, (CI)		15.7 (15.7–15.7)		15.0 (15.0–15.0)		20.3 (20.3–20.4)		22.9 (22.9–22.9)	
p value		0.255		0.007		0.016		0.009	
Female	18–29	18	28.6	20	34.5	17	22.1	22	28.2
	30–44	23	36.5	21	36.2	33	42.8	32	41.0
	45–65	22	34.9	17	29.3	27	35.1	24	30.8
	Total	63	13.9	58	12.8	77	17.0	78	17.2
Age-adjusted %, (CI)		13.9 (13.9–13.9)		12.8 (12.8–12.8)		17.1 (17.1–17.2)		17.3 (17.3–17.3)	
p value		0.999		0.477		0.303		0.590	
Total	18–29	29	27.9	34	36.2	34	26.8	41	30.8
	30–44	32	30.8	28	29.8	44	34.6	46	34.6
	≥ 45	43	41.3	32	34.0	49	38.6	46	34.6
	Total	104	14.6	94	13.2	127	17.8	133	18.6
Age-adjusted %, (CI)		14.8 (14.7–14.8)		13.9 (13.8–13.9)		18.7 (18.6–18.7)		20.0 (19.9–20.1)	
p value		0.631		0.035		0.908		0.212	

* p values were analyzed using the *χ*^2^ test. CI: Confidence interval. ENV: Environmental health domain. PHY: Physical health domain. PSY: Psychological health domain. QOL: Quality of life. SOC: Social relationship domain. The cut-off points were 49 for PHY, 61 for PSY, 54 for SOC, and 54 for ENV.

Table 4 shows the Spearman’s correlation coefficients between the WHOQOL-BREF scores and selected continuous variables. All domains of the WHOQOL-BREF showed a significant inverse correlation with anxiety and depression (p < 0.001), and the coefficients ranged from -0.353 to -0.206 and from -0.335 to -0.156, respectively.

**Table 4:** Spearman correlation coefficients between the WHOQOL-BREF subscale scores and selected factors.

Variables	WHOQOL-BREF domains				Perception
	PHY	PSY	SOC	ENV	GEN
Age	0.020	0.000	-0.062	0.030	-0.013
Body temperature	0.111*	0.008	0.033	0.033	-0.008
Heart rate (per minute)	-0.071	0.006	0.019	-0.012	-0.036
Arterial systolic pressure	-0.022	0.091*	0.021	0.026	0.002
Arterial diastolic pressure	-0.026	0.070	0.003	0.037	0.013
Arterial oxygen saturation	0.007	-0.020	-0.008	-0.039	-0.057
BMI	-0.006	0.014	-0.022	0.001	0.002
Anxiety score	-0.319***	-0.353***	-0.206***	-0.256***	-0.223**
Depression score	-0.321***	-0.335***	-0.249***	-0.257***	-0.156**

* p < 0.05

** p < 0.01

*** p < 0.001. ENV: Environmental health domain. GEN: General facet. PHY: Physical health domain. PSY: Psychological health domain. SOC: Social relationship domain.

Table 5 shows the coefficients of multiple linear regression for each domain of the WHOQOL-BREF. To investigate how QOL was predicted by the sociodemographic characteristics, independent variables were selected for each domain, using a stepwise method. This demonstrates that the physical health domain was predicted by residency location, anxiety, and depression (R2 = 0.200, p < 0.001); psychological health domain by anxiety and depression (R2 = 0.203, p < 0.001); social relatuonship domain by anxiety and depression (R2 = 0.116, p < 0.001); and environmental health domain by sex, waist circumstance, anxiety, and depression (R2 = 0.117, p < 0.001), respectively. The general facet was predicted by age group, residency location, and anxiety (R2 = 0.087, p < 0.001). No multicollinearity was detected between the tested variables; the independence assumption was satisfied; the distribution of the residuals satisfied the normality assumptions. The variance of the model was constant, and homoscedasticity was not violated.

**Table 5:** Multiple linear regression analyses on the WHOQOL-BREF domain scores by demographic characteristics, vital signs, and psychological symptoms.

Variables	B	Beta	t	p value*	95% Confidence Interval for Exp (B)		Collinearity Statistics	
					Lower	Upper	Tolerance	Variance InflationFactor
Physical health domain (R^2^ = 0.200, Durbin-Watson = 2.083, F_18_ = 8.28, p < 0.001)								
Constant	4.24		0.73	0.940	-110.20	118.67		
Residency location (ref: Ulaanbaatar)	-1.86	-0.22	-5.25	< 0.001	-2.55	-1.16	0.77	1.30
Anxiety score	-0.82	-0.21	-4.85	< 0.001	-1.16	-0.49	0.72	1.38
Depression score	-0.90	-0.20	-4.71	< 0.001	-1.28	-0.53	0.74	1.36
Psychological health domain (R^2^ = 0.203, Durbin-Watson = 1.963, F_18_ = 8.448, p < 0.001)								
Constant	106.0		1.89	0.060	-4.31	216.31		
Anxiety score	-1.09	-0.28	-6.64	< 0.001	-1.41	-0.77	0.72	1.38
Depression score	-0.90	-0.21	-4.88	< 0.001	-1.27	-0.54	0.74	1.36
Social relationship domain (R^2^ = 0.116, Durbin-Watson = 1.911, F_18_ = 4.353, p < 0.001)								
Constant	74.40		0.96	0.340	-77.10	225.9		
Age group (ref: 45–65)	-1.89	-0.10	-2.01	0.045	-3.75	-0.04	0.67	1.50
Residency location (ref: Ulaanbaatar)	-1.10	-0.10	-2.35	0.019	-2.02	-0.18	0.77	1.30
Anxiety score	-0.65	-0.13	-2.89	0.004	-1.09	-0.21	0.72	1.38
Depression score	-1.17	-0.21	-4.59	< 0.001	-1.67	-0.67	0.74	1.36
Environmental health domain (R^2^ = 0.117, Durbin-Watson = 2.000, F_18_ = 4.393, p < 0.001)								
Constant	26.38		0.41	0.682	-100.21	152.97		
Employment (ref: employed)	1.25	0.10	2.20	0.027	0.04	2.36	0.70	1.42
Anxiety score	-0.87	-0.21	-4.60	< 0.001	-1.23	-0.50	0.72	1.38
Depression score	-0.67	-0.14	-3.10	0.002	-1.08	-0.25	0.74	1.36
General facet (R^2^ = 0.087, Durbin-Watson = 1.993, F_18_ = 3.142, p < 0.001)								
Constant	118.55		1.59	0.113	-28.22	265.31		
Age group (ref: 45–65)	-2.01	-0.11	-2.20	0.028	-3.81	-0.22	0.67	1.50
Residency location (ref: Ulaanbaatar)	-0.98	-0.10	-2.16	0.031	-1.87	-0.09	0.77	1.30
Anxiety score	-0.98	-0.21	-4.51	< 0.001	-1.41	-0.56	0.72	1.38

* p values were tested using the multiple linear regression. Factors: (constant), sex, age group, marital status, education, employment, income, living condition, residency location, alcohol and tobacco use, body temperature, heart rate, systolic blood pressure, diastolic blood pressure, arterial oxygen saturation, body mass index, anxiety and depression score. ref: A reference group for categorical variables.

**Fig 2:**
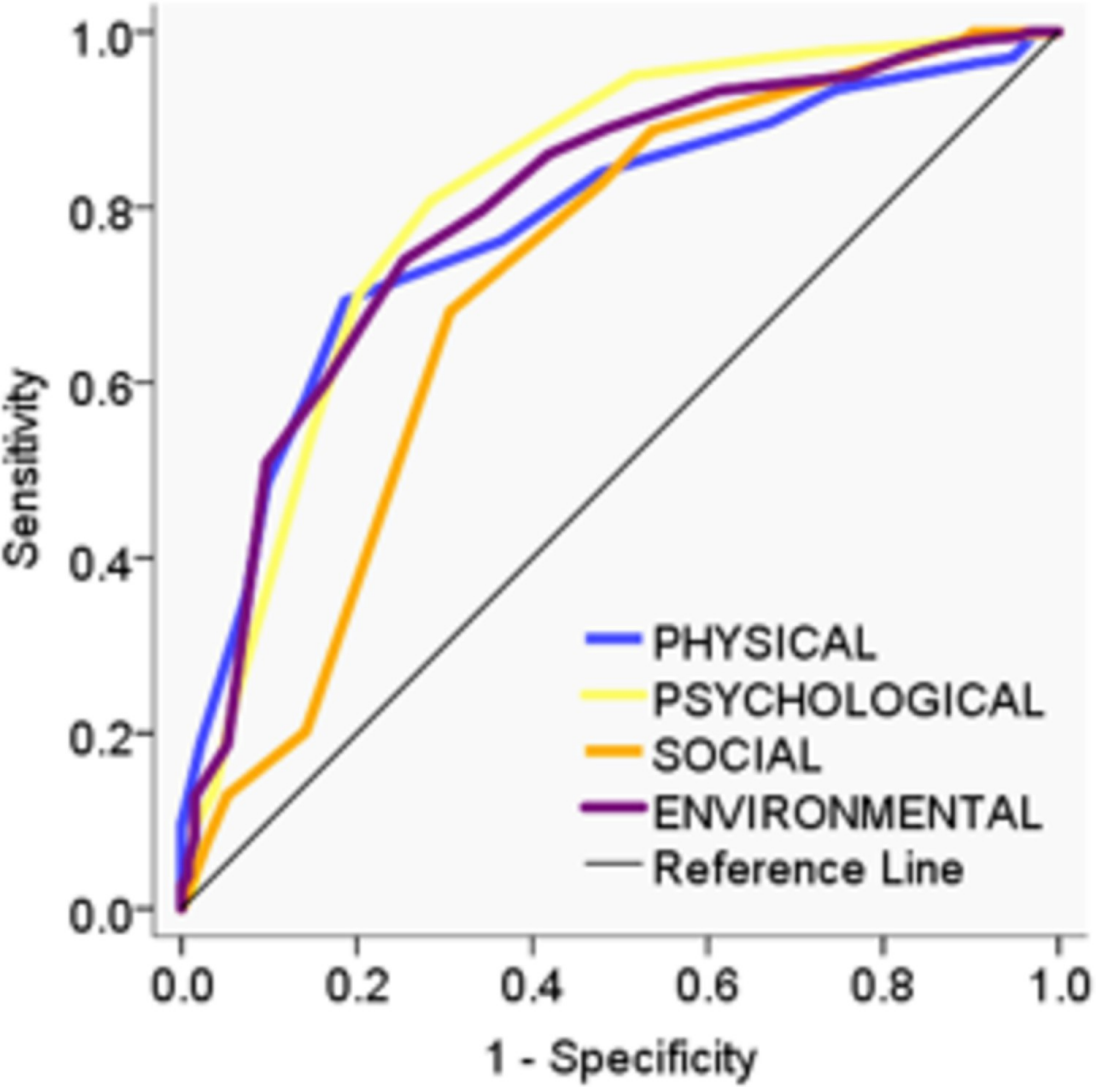
ROC curve of the WHOQOL-BREF domains. AUC values were 0.78, 0.81, 0.71, and 0.80 for the physical health, psychological health, social relationship, and environmental health domains, respectively.

As described in the Methods section, we demonstrated the true-positive rates of each domain against the false-positive rate at a variety of thresholds for a single variable created by dichotomizing the general facet at 1 SD below the mean score (Fig 2).

The area under the ROC curve (AUC) indicates performance; the greater the AUC, the better the performance. Four cut-off points were set to calculated sensitivity, specificity, positive predictive value, negative predictive value, and Youden’s index for each domain (S4 Table); 1 SD below the mean, 95% of sensitivity, 95% of specificity, and the highest of Youden’s index. Youden’s index was the highest for psychological health domain (0.52) and the lowest for social relationship domain (0.38).

## Discussion

This is the first report on the normative data on QOL in the general population of Mongolia using the WHOQOL-BREF. The WHOQOL-BREF is a frequently used self-report questionnaire for QOL which has more merits than shortcomings [16]. In our previous study, we validated the Mongolian version of the WHOQOL-BREF, which showed good validity and reliability for assessing QOL [8]. However, we assessed the questionnaire’s psychometric properties only in the general urban population. The present study investigated the differences in QOL between urban and rural residents. The sample population was chosen to represent the target population, considering sociodemographic characteristics. The level of internal consistency was acceptable to good. Therefore, the current results provide valuable information on QOL in the general population of Mongolia.

The present findings suggest that the Mongolian adults in this sample had a specific QOL pattern in terms of domain characteristics of WHOQOL-BREF. As shown in Table 6, the physical health domain score was lower, whereas the psychological health, social relationship, and environmental health domain scores were higher or similar compared with those of other countries [12,16–20]. Furthermore, the psychological health was relatively higher compared to other countries except Norway.

**Table 6:** WHOQOL-BREF domain scores by different countries.

Countries	n	PHY	PSY	SOC	ENV
Mongolia (this study) ^a^	714	13.8±2.0	15.8±1.9	15.3±2.5	14.8±2.1
Mongolia (this study) ^b^		61.5±12.6	73.6±12.1	70.4±15.9	67.6±13.4
Norway (Kafloss et al. 2021) [17]^a^	615–626	16.5±2.6	15.9±2.2	15.4±2.6	15.3±2.2
Pakistan (Lodhi et al. 2019) [12] ^b^	2063	65.2±15.2	67.4±15.0	72.0±16.5	55.5±15.0
China (Xia et al. 2012) [18] ^a^	1052	14.6±2.0	13.7±2.2	14.1±2.2	12.3±2.3
China (Xia et al. 2012) [18] ^b^		66.0±12.6	60.6±14.0	63.2±13.9	52.0±14.5
Iran (Nedjat et al. 2008) [19] ^a^	906	14.7±2.3	13.7±2.5	14.1±2.4	12.7±2.4
Taiwan (Wang et al. 2006) [20] ^b^	13083	59.1±13.7	49.4±15.6	56.5±14.3	42.4±14.9
Global (Skevington et al. 2002: 23 countries) [16] ^a^	11830	16.2±2.9	15.0±2.1	14.3±3.2	13.5±2.6

^a^ scale in the range 4–20. ^b^ scale in the range 1–100. ENV: Environmental health domain; PHY: Physical health domain; PSY: Psychological health domain; SOC: Social relationship domain. n: Number.

By the estimated prevalence rates of good/poor QOL, the Mongolian adults showed a relatively higher proportion of poor QOL (ranging from 13 to 19%) compared with Chinese adults (ranging from 7 to 16%) [18]. These findings might be reasonable because the life expectancy of the Mongolians is still low compared with that of developed countries. In addition, we found that living in an apartment in an urban area had a negative influence on physical health. It might be associated with the recent rapid urbanization that can cause decreased physical activity, implicating obesity. Psychological problems, including anxiety and depression, had a strong negative impact on each domain of the WHOQOL-BREF. A high rate of unemployment, low income, increased alcohol consumption, and living in urban areas were associated with the underlying high rate of anxiety and depression. These findings also support our previous studies that measured mental distress and sleep quality in the general population [21,22]. Employment was directly related to an increased QOL. Sex did not affect the QOL, and there was a tendency for male participants to have lower environmental QOL than female participants. Unlike the other Asian cultures, Mongolia does not share male dominance in society. Age was an important factor for social QOL, which declined in older participants. Living in urban areas has harmed the physical and social domain. Because Ulaanbaatar is the coldest capital and one of the most air-polluted cities in the world, many health issues have been addressed. Moreover, the intentional homicide rate in Mongolia was among the highest in Asian countries in the last few decades after Iraq [23]. In clinical implication, the findings of this nationwide population-based study seem to provide comprehensive information for evidence-based policy and service quality assessment for health care.

The main limitations of this study are as follows: (i) as a self-report questionnaire, WHOQOL-BREF is based on subjective answers; (ii) although this study investigated the QOL in the adult population using WHOQOL-BREF, future studies should include more tools such as EQ-5D and SF-36 to determine criterion validity. Despite these limitations, we believe this is the first study to provide normative data for the WHOQOL-BREF in Mongolia, enabling further studies to use the results as baseline data and compare them with international findings.

## Conclusion

This is the first report on normative data on the QOL in the general population of Mongolia. Physical health was low compared with that of international data. Poor QOL was observed among those living in urban areas with mental health issues.

## Supporting information

S1 Table The number and percentage of the sample in each age-sex category compared with results from the 2020 Population and Housing By-Census of Mongolia. (DOCX)

S2 Table Normative values of the WHOQOL-BREF scores by living conditions. (DOCX)

S3 Table Normative values of the WHOQOL-BREF scores by residency locations. (DOCX)

S4 Table Cut-off scores of the WHOQOL-BREF domains. (DOCX)
