# Supplementary material for: Quality of life in the general population of Mongolia: Normative data on WHOQOL-BREF
Source: PLoS One. 2023 Sep 29;18(9):e0291427. doi: 10.1371/journal.pone.0291427 (PMC10540971; doi:10.1371/journal.pone.0291427)
Supplement: S1 Table — (DOCX) [file pone.0291427.s001.docx]

**S1 Table. The number and percentage of the sample in each age-sex category compared with results from the 2020 Population and Housing By-Census of Mongolia.**

| **Age-sex category** | | **Sample size, n (%)** | | **Population, n (%)** | |
| --- | --- | --- | --- | --- | --- |
| Total | | 714 | (100) | 1910630 | (100) |
| Both sex | 18-29 | 181 | (25.4) | 610322 | (31.9) |
|  | 30-44 | 248 | (34.7) | 780603 | (40.9) |
|  | 45-65 | 285 | (39.9) | 519705 | (27.2) |
| Male | 18-29 | 52 | (7.3) | 273476 | (14.3) |
|  | 30-44 | 83 | (11.6) | 364514 | (19.1) |
|  | 45-65 | 126 | (17.7) | 282314 | (14.8) |
| Female | 18-29 | 129 | (18.1) | 270846 | (14.2) |
|  | 30-44 | 165 | (23.1) | 385836 | (20.2) |
|  | 45-65 | 159 | (22.2) | 333644 | (17.4) |
| Residence location | Ulaanbaatar city | 312 | (43.7) | 860488 | (45.0) |
|  | Eastern region | 84 | (11.8) | 163870 | (8.7) |
|  | Western region | 107 | (15.0) | 252805 | (13.2) |
|  | Mountain region | 110 | (15.4) | 340649 | (17.8) |
|  | Central region | 101 | (14.1) | 292818 | (15.3) |

n: number.
