# Supplementary material for: Quality of life in the general population of Mongolia: Normative data on WHOQOL-BREF
Source: PLoS One. 2023 Sep 29;18(9):e0291427. doi: 10.1371/journal.pone.0291427 (PMC10540971; doi:10.1371/journal.pone.0291427)
Supplement: S2 Table — (DOCX) [file pone.0291427.s002.docx]

**S2 Table. Normative values of the WHOQOL-BREF scores by living conditions.**

| **Characteristics (n)** | | **WHOQOL domains (mean ± SD)** | | | | **Perception** |
| --- | --- | --- | --- | --- | --- | --- |
|  |  | **PHY** | **PSY** | **SOC** | **ENV** | **GEN** |
| Total | | 61.47 | 73.55 | 70.38 | 67.60 | 70.47 |
| **Ger (no public utilities)** | | 62.17 | 74.16 | 71.18 | 68.63 | 70.39 |
| Age group | 18-29 | 60.31 | 72.19 | 71.16 | 68.68 | 69.66 |
|  | 30-44 | 63.15 | 74.80 | 72.80 | 68.75 | 71.11 |
|  | 45-65 | 62.27 | 74.58 | 69.97 | 68.51 | 70.18 |
| Sex | Male | 62.51 | 75.16 | 71.48 | 68.83 | 70.65 |
|  | Female | 61.97 | 73.58 | 71.00 | 68.51 | 70.24 |
| Marital status | Never-married | 60.97 | 73.21 | 70.68 | 67.19 | 71.88 |
|  | Others* | 63.79 | 76.50 | 73.50 | 71.38 | 72.00 |
|  | Married | 62.13 | 73.97 | 70.91 | 68.45 | 69.89 |
| Education | Middle school and below | 63.21 | 75.26 | 71.74 | 69.46 | 70.78 |
|  | Associate’s degree | 62.53 | 73.45 | 72.25 | 68.14 | 69.49 |
|  | Bachelor’s degree | 59.36 | 72.66 | 68.39 | 67.57 | 70.88 |
|  | Master’s degree and above | 57.14 | 69.91 | 68.52 | 63.89 | 68.06 |
| Employment | Unemployed | 61.22 | 74.09 | 71.74 | 68.36 | 70.70 |
|  | Student | 59.77 | 71.08 | 69.36 | 64.43 | 69.49 |
|  | Pensioner | 62.96 | 73.82 | 69.20 | 68.11 | 70.96 |
|  | Employed | 62.44 | 74.79 | 72.15 | 69.54 | 70.19 |
| Income | < ₮500,000 | 62.45 | 74.25 | 71.07 | 68.73 | 70.99 |
|  | ₮500,001 - ₮1,000,000 | 61.36 | 73.85 | 71.13 | 68.16 | 69.00 |
|  | > ₮1,000,001 | 64.73 | 76.04 | 76.04 | 72.27 | 70.31 |
| Residence location | Rural areas | 64.14 | 74.56 | 71.33 | 68.98 | 70.86 |
|  | Urban areas | 58.08 | 73.35 | 70.86 | 67.89 | 69.41 |
| Alcohol use | Yes | 61.41 | 74.56 | 69.58 | 68.33 | 69.66 |
|  | No | 62.36 | 74.02 | 71.73 | 68.73 | 70.64 |
| Tobacco use | Yes | 64.00 | 75.51 | 73.87 | 70.82 | 71.62 |
|  | No | 61.95 | 73.86 | 70.74 | 68.13 | 70.50 |
|  | Had smoked before | 56.79 | 73.33 | 66.67 | 68.44 | 61.25 |
| **Apartment (with public utilities)** | | 60.40 | 72.60 | 69.14 | 66.02 | 70.58 |
| Age group | 18-29 | 62.11 | 71.69 | 69.66 | 66.58 | 74.05 |
|  | 30-44 | 60.43 | 74.21 | 70.17 | 65.81 | 68.13 |
|  | 45-65 | 58.56 | 71.73 | 67.42 | 65.66 | 69.74 |
| Sex | Male | 59.82 | 71.29 | 66.92 | 62.50 | 72.13 |
|  | Female | 60.71 | 73.33 | 70.37 | 67.97 | 69.72 |
| Marital status | Never-married | 59.98 | 69.18 | 66.14 | 65.23 | 72.22 |
|  | Others* | 60.16 | 74.20 | 71.15 | 66.47 | 66.35 |
|  | Married | 60.56 | 73.52 | 69.85 | 66.21 | 70.62 |
| Education | Middle school and below | 60.48 | 70.73 | 67.53 | 64.08 | 73.21 |
|  | Associate’s degree | 60.24 | 74.33 | 70.10 | 67.37 | 71.32 |
|  | Bachelor’s degree | 60.20 | 71.81 | 68.02 | 65.82 | 68.24 |
|  | Master’s degree and above | 61.46 | 77.43 | 76.74 | 69.27 | 70.83 |
| Employment | Unemployed | 58.18 | 69.79 | 67.71 | 61.33 | 68.75 |
|  | Student | 64.00 | 74.92 | 72.17 | 68.81 | 79.25 |
|  | Pensioner | 56.58 | 69.52 | 65.35 | 63.65 | 67.11 |
|  | Employed | 60.50 | 73.02 | 69.30 | 66.39 | 69.05 |
| Income | < ₮500,000 | 59.21 | 71.57 | 67.59 | 64.88 | 71.39 |
|  | ₮500,001 - ₮1,000,000 | 61.72 | 73.44 | 70.87 | 67.39 | 70.32 |
|  | > ₮1,000,001 | 59.44 | 74.70 | 67.86 | 64.06 | 65.18 |
| Residency location | Rural areas | 63.17 | 74.24 | 71.79 | 66.66 | 72.94 |
|  | Urban areas | 58.63 | 71.56 | 67.45 | 65.61 | 69.08 |
| Alcohol use | Yes | 59.78 | 71.93 | 68.85 | 65.23 | 67.66 |
|  | No | 60.18 | 72.64 | 69.10 | 66.63 | 71.27 |
| Tobacco use | Yes | 59.97 | 70.52 | 67.30 | 65.92 | 69.81 |
|  | No | 60.26 | 73.28 | 69.78 | 66.81 | 70.19 |
|  | Had smoked before | 59.92 | 68.52 | 68.52 | 61.46 | 73.61 |

p < 0.05, p < 0.01, p < 0.001. * Others included remarried, co-habiting, separated, divorced, and widowed. ENV: environmental health domain. GEN: general facet. PHY: physical health domain. PSY: psychological health domain. SOC: social relationship domain. There were no missing data, except for 91 and 108 participants who did not report alcohol and tobacco use, respectively.
