# Supplementary material for: Quality of life in the general population of Mongolia: Normative data on WHOQOL-BREF
Source: PLoS One. 2023 Sep 29;18(9):e0291427. doi: 10.1371/journal.pone.0291427 (PMC10540971; doi:10.1371/journal.pone.0291427)
Supplement: S3 Table — (DOCX) [file pone.0291427.s003.docx]

**S3 Table. Normative values of the WHOQOL-BREF scores by residency locations.**

| **Characteristics (n)** | | **WHOQOL domains (mean ± SD)** | | | | **Perception** |
| --- | --- | --- | --- | --- | --- | --- |
|  |  | **PHY** | **PSY** | **SOC** | **ENV** | **GEN** |
| Total | | 61.47 | 73.55 | 70.38 | 67.60 | 70.47 |
| **Rural areas** | | 63.88 | 74.47 | 71.46 | 68.35 | 71.42 |
| Age group | 18-29 | 63.15 | 73.52 | 71.79 | 68.18 | 74.40 |
|  | 30-44 | 64.61 | 76.35 | 73.87 | 69.38 | 72.37 |
|  | 45-65 | 63.74 | 73.56 | 69.29 | 67.63 | 68.79 |
| Sex | Male | 63.50 | 74.54 | 70.45 | 67.41 | 72.08 |
|  | Female | 64.11 | 74.43 | 72.08 | 68.94 | 71.02 |
| Marital status | Never-married | 62.50 | 71.02 | 69.49 | 65.07 | 73.35 |
|  | Others* | 65.77 | 76.62 | 74.31 | 71.44 | 70.83 |
|  | Married | 63.96 | 75.00 | 71.56 | 68.73 | 71.06 |
| Education | Middle school and below | 63.67 | 74.38 | 71.46 | 68.34 | 72.02 |
|  | Associate’s degree | 64.93 | 75.54 | 72.33 | 68.19 | 71.38 |
|  | Bachelor’s degree | 63.10 | 72.44 | 69.34 | 67.99 | 69.55 |
|  | Master’s degree and above | 63.93 | 81.67 | 79.17 | 73.13 | 73.75 |
| Employment | Unemployed | 64.37 | 74.90 | 71.67 | 68.59 | 74.69 |
|  | Student | 62.68 | 73.30 | 72.30 | 67.30 | 75.70 |
|  | Pensioner | 64.68 | 73.46 | 68.83 | 67.55 | 68.83 |
|  | Employed | 63.88 | 75.18 | 72.14 | 68.97 | 70.36 |
| Income | < ₮500,000 | 63.72 | 74.04 | 70.80 | 68.24 | 72.16 |
|  | ₮500,001 - ₮1,000,000 | 64.48 | 75.46 | 73.15 | 68.92 | 70.44 |
|  | > ₮1,000,001 | 61.01 | 73.61 | 68.06 | 64.84 | 65.63 |
| Living condition | Ger (no public utilities) | 64.14 | 74.56 | 71.33 | 68.98 | 70.86 |
|  | Apartment (with public utilities) | 63.17 | 74.24 | 71.79 | 66.66 | 72.94 |
| Alcohol use | Yes | 62.79 | 74.28 | 71.68 | 67.73 | 70.54 |
|  | No | 64.40 | 74.62 | 71.64 | 68.87 | 72.02 |
| Tobacco use | Yes | 65.91 | 75.29 | 71.05 | 70.18 | 73.03 |
|  | No | 63.79 | 74.42 | 71.76 | 68.18 | 71.29 |
|  | Had smoked before | 59.42 | 71.21 | 74.24 | 69.03 | 73.86 |
| **Urban areas (Ulaanbaatar)** | | 58.38 | 72.37 | 68.99 | 66.64 | 69.23 |
| Age group | 18-29 | 58.63 | 69.81 | 68.51 | 66.84 | 68.51 |
|  | 30-44 | 59.10 | 72.50 | 69.28 | 65.46 | 67.07 |
|  | 45-65 | 57.53 | 73.89 | 69.03 | 67.63 | 71.77 |
| Sex | Male | 58.58 | 72.43 | 68.69 | 64.95 | 69.98 |
|  | Female | 58.28 | 72.34 | 69.15 | 67.52 | 68.84 |
| Marital status | Never-married | 57.70 | 71.16 | 66.67 | 67.59 | 70.34 |
|  | Others* | 59.64 | 74.90 | 71.25 | 68.13 | 69.38 |
|  | Married | 58.31 | 72.19 | 69.12 | 66.15 | 68.95 |
| Education | Middle school and below | 59.70 | 73.44 | 68.75 | 67.47 | 69.89 |
|  | Associate’s degree | 57.93 | 71.71 | 70.45 | 67.48 | 68.75 |
|  | Bachelor’s degree | 57.64 | 71.99 | 67.39 | 65.60 | 69.24 |
|  | Master’s degree and above | 58.70 | 72.64 | 72.46 | 65.49 | 68.48 |
| Employment | Unemployed | 57.07 | 71.27 | 69.79 | 64.65 | 66.41 |
|  | Student | 60.16 | 73.72 | 64.10 | 65.63 | 73.08 |
|  | Pensioner | 56.48 | 71.54 | 67.23 | 66.00 | 71.40 |
|  | Employed | 59.17 | 72.81 | 69.66 | 67.40 | 69.01 |
| Income | < ₮500,000 | 57.90 | 72.45 | 68.73 | 66.43 | 69.49 |
|  | ₮500,001 - ₮1,000,000 | 58.74 | 71.91 | 68.94 | 66.67 | 68.94 |
|  | > ₮1,000,001 | 61.79 | 77.08 | 74.17 | 69.69 | 68.75 |
| Living condition | Ger (no public utilities) | 58.08 | 73.35 | 70.86 | 67.89 | 69.41 |
|  | Apartment (with public utilities) | 58.63 | 71.56 | 67.45 | 65.61 | 69.08 |
| Alcohol use | Yes | 58.28 | 72.40 | 66.47 | 66.10 | 66.76 |
|  | No | 57.98 | 72.11 | 69.65 | 66.81 | 69.43 |
| Tobacco use | Yes | 59.39 | 71.90 | 71.19 | 67.63 | 69.11 |
|  | No | 57.82 | 72.55 | 68.43 | 66.85 | 69.10 |
|  | Had smoked before | 56.70 | 70.83 | 58.33 | 59.77 | 57.81 |

p < 0.05. * Others included remarried, co-habiting, separated, divorced, and widowed. ENV: environmental health domain. GEN: general facet. PHY: physical health domain. PSY: psychological health domain. SOC: social relationship domain. There were no missing data, except for 91 and 108 participants who did not report alcohol and tobacco use, respectively.
