# Supplementary material for: Quality of life in the general population of Mongolia: Normative data on WHOQOL-BREF
Source: PLoS One. 2023 Sep 29;18(9):e0291427. doi: 10.1371/journal.pone.0291427 (PMC10540971; doi:10.1371/journal.pone.0291427)
Supplement: S4 Table — (DOCX) [file pone.0291427.s004.docx]

**S4 Table. Cut-off scores of the WHOQOL-BREF domains.**

| **Domains** | **Cut-point** | | **Sensitivity** | **Specificity** | **PPV** | **NPV** | **J** |
| --- | --- | --- | --- | --- | --- | --- | --- |
| PHY  AUC: 0.78 | < Mean – 1 SD | 49 | 88% | 32% | 84% | 41% | 0.20 |
|  | > 95% Sensitivity | 43 | 96% | 10% | 82% | 38% | 0.06 |
|  | > 95% Specificity | 75 | 19% | 98% | 97% | 22% | 0.17 |
|  | Maximum Youden’s index | 61 | 69% | 81% | 94% | 38% | 0.51 |
| PSY  AUC: 0.81 | < Mean – 1 SD | 61 | 96% | 47% | 88% | 71% | 0.37 |
|  | > 95% Sensitivity | 63 | 95% | 49% | 89% | 69% | 0.44 |
|  | > 95% Specificity | 88 | 13% | 96% | 94% | 20% | 0.10 |
|  | Maximum Youden’s index | 71 | 81% | 72% | 92% | 46% | 0.52 |
| SOC  AUC: 0.71 | < Mean – 1 SD | 54 | 95% | 43% | 84% | 57% | 0.25 |
|  | > 95% Sensitivity | 50 | 97% | 19% | 84% | 57% | 0.15 |
|  | > 95% Specificity | 92 | 13% | 95% | 91% | 20% | 0.08 |
|  | Maximum Youden’s index | 75 | 68% | 69% | 91% | 33% | 0.38 |
| ENV  AUC: 0.80 | < Mean – 1 SD | 54 | 91% | 44% | 88% | 58% | 0.35 |
|  | > 95% Sensitivity | 50 | 95% | 23% | 84% | 52% | 0.18 |
|  | > 95% Specificity | 78 | 19% | 95% | 94% | 21% | 0.13 |
|  | Maximum Youden’s index | 66 | 74% | 76% | 93% | 40% | 0.49 |

AUC: area under the curve. ENV: environmental health domain. J: the highest value of Youden’s index. NPV: negative predictive value. PHY: physical health domain. PPV: positive predictive value. PSY: psychological health domain. SD: standard deviation. SOC: social relationship domain. n: number.
